# Socioeconomic position and self-rated health among adolescents: the mediating role of the family

**DOI:** 10.1093/eurpub/ckac129.580

**Published:** 2022-10-25

**Authors:** P Rattay, M Blume, J Spallek, S Hoffmann, L Sander, R Herr, M Herke, M Reuter, A Novelli, C Hövener

**Affiliations:** 1 Robert Koch-Institute, Department of Epidemiology and Health Monitoring, Berlin, Germany; 2 Brandenburg University of Technology, Department of Public Health, Senftenberg, Germany; 3 Heidelberg University, Center for Preventive Medicine and Digital Health, Mannheim, Germany; 4 Martin Luther University Halle-Wittenberg, Institute of Medical Sociology, Halle, Germany; 5 Heinrich Heine University Düsseldorf, Institute of Medical Sociology, Düsseldorf, Germany; 6 Technical University of Munich, Department of Sport and Health Sciences, Munich, Germany

## Abstract

**Background:**

Although health inequalities in adolescence are well documented, the underlying mechanisms remain unclear. Few studies have examined the role of the family in explaining adolescents’ health inequalities. The study aimed to explore whether the association between socioeconomic position and self-rated health (SRH) was mediated by familial determinants.

**Methods:**

Using data from wave 2 of the KiGGS study (1,838 female and 1,718 male 11- to 17-year-olds), linear regression analyses were conducted to decompose the total effects of parents’ education, occupation, income, socioeconomic position index, and adolescents’ subjective social status on SRH into direct effects and indirect effects through familial determinants (family cohesion, parenting styles, parental well-being, stress, obesity, smoking and sporting activity).

**Results:**

A significant total effect of all socioeconomic position indicators on SRH was found, except for income in male adolescents. In female adolescents, more than 70% of the total effects of each socioeconomic position indicator were explained by familial mediators, whereas no significant direct effects remained. The most important mediator was parental well-being, followed by family cohesion, parental smoking and sporting activity. In male adolescents, the associations of parental education, the socioeconomic position index and subjective social status with SRH were also mediated by familial determinants (family cohesion, parental smoking and obesity). However, a significant direct effect of subjective social status remained.

**Conclusions:**

The family appears to play an important role in explaining health inequalities, particularly in female adolescents. Reducing health inequalities in adolescence requires policy interventions, community-based strategies, as well as programs to improve parenting and family functioning.

